# Association between brain-derived neurotropic factor (BDNF), high-sensitivity C-reactive protein (hs-CRP) and psychiatric symptoms in medicated and unmedicated patients

**DOI:** 10.1186/s12888-022-03744-2

**Published:** 2022-02-03

**Authors:** Hedda Soloey-Nilsen, Kristin Nygaard-Odeh, Magnhild Gangsoey Kristiansen, Ole Lars Brekke, Tom Eirik Mollnes, Solveig Klaebo Reitan, Terje Oiesvold

**Affiliations:** 1 Nordland Hospital Trust, N-8092 Bodø, Norway; 2 Institute of Clinical Medicine, UIT The Arctic University of Norway, Tromsø, Norway; 3 Research Laboratory, Nordland Hospital Trust, and, Faculty of Health Sciences, K.G. Jebsen TREC, University of Tromsø, Tromsø, Norway; 4 Department of Immunology, Oslo University Hospital and University of Oslo, Oslo, Norway; 5 Centre of Molecular Inflammation Research, Norwegian University of Science and Technology, Trondheim, Norway; 6 Department of Mental Health IPH, Faculty of Medicine and Health sciences, Norwegian University of Science and Technology, NTNU, Trondheim, Norway

**Keywords:** Brain derived neurotropic factor (BDNF), high-sensitive- C-reactive protein (hs-CRP), general psychiatric symptoms

## Abstract

**Background:**

There is evidence that brain-derived neurotropic factor (BDNF) plays a protective role in the brain. Peripheral levels of BDNF correlate with its concentration in the brain. Previous studies have revealed lower serum BDNF levels in patients with mental illnesses. In most studies serum BDNF correlates negatively with psychiatric disorders and disease severity. Most studies in this field are on psychiatric diagnosis and personality traits. The aim of our study is to explore associations between general psychiatric symptoms, independent of diagnostic groups, and serum BDNF as well as the inflammatory biomarker high-sensitive CRP (hs-CRP). Comparison between the group regularly using psychotropic medication and those not using psychotropic medication is conducted.

**Methods:**

The study is a cross sectional study with 132 participants from a general open inpatient psychiatric ward at the Nordland Hospital Trust, Bodoe, Norway. Participants were assessed on serum levels of BDNF and hs-CRP. Psychiatric symptoms were assessed by a self-rating scale (Symptom check list, SCL-90- R). Multiple linear regression model was used for statistical analyses of associations between levels of BDNF, hs-CRP and symptoms.

**Results:**

We found a positive association (*p* < 0.05), for most SCL-90 symptom clusters with BDNF in the psychotropic medication-free group. No associations were found in the group of patients using psychotropic medication, except one, the paranoid ideation cluster (*p* 0.022). No associations were found between hs-CRP and symptom clusters.

**Conclusion:**

We found no relation between symptom clusters and the inflammatory biomarker hs-CRP. Serum BDNF levels were positively associated with intensity of psychiatric symptoms in the group of patients not using psychotropic medication. Our findings are in conflict with several previous studies reporting increased hs-CRP as well as decreased rather than increased BDNF in mental suffering. Patients on psychotropic medication may not require the same upregulation because the medication is modulating the underlying biological pathology.

## Introduction

Identifying the causative and maintaining role of biochemical factors in mental illness may aid in the development of more effective treatment and prevention of mental illness. Brain derived neurotrophic factor (BDNF) is a widely distributed substance in the central nervous system. It plays an important role in the adult brain, regulating neuronal integrity, promoting synaptic plasticity. It also modulates synthesis, metabolism and release of neurotransmitters. Thus, BDNF may be important in the pathology of mental suffering like depression. BDNF crosses the blood brain barrier (BBB) and serum concentrations strongly correlate with brain levels [1–4].

Animal models demonstrate stress-induced dysregulation of BDNF-expression, especially in response to chronic stress [5]. In humans, lower serum BDNF levels have been seen in patients with major depressive disorder (MDD), bipolar disorder (BD), schizophrenia, eating disorder, obsessive-compulsive disorder (OCD) and alcohol dependency [5]. In most studies involving MDD, serum BDNF levels correlated negatively with disease severity, and antidepressant treatment increased serum levels of BDNF, as do antipsychotics in schizophrenia [6]. These findings could indicate that a low BDNF level reflects general mental stress. Some studies found that serum BDNF levels were not influenced by neither type of psychotropic medication, nor doses. They found no significant association between antidepressants and BDNF levels, but recommended follow-up studies in drug-naïve patient [5]. Most studies in this field are, however, on distinct diagnostic groups or personality traits compared to healthy controls [5]. These diagnostic groups may include a heterogeneous spectre of symptoms and a specific role of BDNF in specific symptoms has not been described.

C-reactive protein (CRP) is a well-known biological marker of systemic inflammation. High sensitivity (hs)-CRP is a more sensitive test for subtle inflammation, and serum hs-CRP may reflect a low-grade systemic inflammatory state of various mental disorders [7]*.* CRP is synthesized in the liver in response to certain pro-inflammatory cytokines [8].

As the psychiatric diagnostic groups are merely syndromal, with collections of symptoms or signs often seen together, they may not represent one common biological process. The diagnostic groups for mental illnesses are wide and partly unspecific, masking differences within the groups [9]. Also, the observed rate of co-occurrence of symptoms among some general psychiatric disorders is high [10], and could in part be due to their multi-factorial nature [11]. Mental suffering and symptoms may also be grouped into “clusters of symptoms” independent of diagnostic groups. One of the most widely replicated disorders are the mood and anxiety disorders [12].

Studying symptoms in relation to biological markers as possible indicators of disease or symptoms may be crucial to reveal causative relations. There are a few reports on altered levels of biological markers in relation to specific symptoms rather than diagnostic groups, e.g. hs-CRP and MCP in fatigue [13] and TGF and aggression / agitation [14], but this field is not extensively studied. To the best of our knowledge, no studies have so far investigated the relation between psychiatric symptoms in patients with known mental disorders and serum BDNF as well as hs-CRP.

The aim of the present study was to explore the associations between psychiatric symptoms as measured by of SCL-90-R with serum BDNF and hs-CRP levels in a patient sample admitted to open ward inpatient stay for treatment of psychiatric disorders. Also, we wanted to compare the group regularly using psychotropic medication and those not using psychotropic medication.

## Methods

### Participants

In this cross-sectional study a total of 138 patients were recruited from an open in-patient general psychiatric ward, at the Department of Mental health and Addiction, Nordland Hospital Trust, Bodoe, Norway. Six participants were excluded due to incomplete data sets.

Patients aged 18 years and above were recruited consecutively in the period February 2014 to February 2018. Patients were referred from the hospital’s outpatient services and from general practitioners. Patients not giving their consent and not understanding Norwegian language or otherwise unable to give informed consent were excluded. A research nurse informed eligible patients about the study and written informed consent was obtained by a doctor administering the clinical assessments.

### Ethics

The study was approved by the Regional Ethics Committee (notification 2015/1809/REK Nord).

### Data collection and assessment of psychiatric symptoms

The following data were obtained from patients: Weight, height and smoking habit.

Age and gender were given from person identification data. All the patients were assessed by the main investigator (first author) upon consultation approximately one week after admittance to the ward. At assessment a rating scale for psychiatric symptoms was administered. Symptom check list 90 (SCL-90-R) is a validated 90 item rating scale used in research for monitoring actual symptoms and symptom clusters experienced by the patient during the last week. SCL-90 gives a valid picture of mental suffering, and is also considered a useful tool for measuring psychological status, measuring change in outcome studies, or screening for mental disorders. Each of the 90 items is rated on a five-point scale, ranging from, not at all (0) to extremely (4) [15]. The 90 single items are often grouped as primary dimensions or clusters: Depression, somatization, obsessive compulsive, interpersonal sensitivity, anxiety, anger hostility, phobic anxiety, paranoid ideation, and psychoticism. All symptom clusters are measured with raw scores in our investigation. Global severity index (GSI) is an index that provide measures of overall psychological distress. We stratified the sample in two subgroups: Those using psychotropic medication regularly and those not using psychotropic medication.

### Biological measures

Blood was withdrawn by trained technicians in the morning the day of assessment between 08:00-10:00 a.m., after approximately 12 hours of fasting and rest and no exercise. Biochemical measures were performed at the Department of Laboratory Medicine, Nordland Hospital Trust. For measurement of serum BDNF, blood was withdrawn in Vacuette gel-tubes, left for 30 minutes on ice before centrifugation for 10 minutes at 2300 x g (3500 rpm). Serum (2 x 1 mL) was stored in Matrix tubes on ice up to 2 hours before freezing at -80^o^ C. Human Free BDNF Quantikine ELISA Kit (R&D Systems, Minneapolis, MN, USA) was used according to the instructions from the manufacturer and performed by trained technicians. hs-CRP in serum was analyzed on a Siemens Prospec nephelometer using CardioPhase® hs-CRP reagents kits from Siemens Healthineers (Erlangen, Germany).

### Statistical analysis

Multiple linear regression analysis was performed using serum BDNF and hs-CRP as dependent variables and symptom clusters as well as confounding factors as BMI, smoking, age and sex as independent variables. The sample were split into two subgroups; patient using psychotropic medication regularly and patients not using psychotropic medication. For all analyses the IBM-SPSS version 26.0 was used, and the statistical significance was set at *p* < 0.05.

## Results

### Demographics

The characteristics of participants are presented in Table 1. Among the 132 patients there were 84 women and 48 men with a mean age of 37 years. Patients were recruited over a 4-year period. A total of 102 patients of the 132 were using psychotropic medication.

**Table 1:** Characteristics of study participants

	n	%		
Gender (male)	47	36		
Smoking	60	46		
Psychotropic medication	102	77		
Antidepressant	87	66		
Antipsychotic	5	4		
Other	10	8		
	Min	Max	Mean	SD
BMI	15	55	28	7
Age (years) BDNF (pg/ml) hs-CRP	18 13093 0.01	78 50435 14	37 27184 2.7	14 6253 3

BDNF: Normal range: 6186-42580 pg/mL. Hs-CRP: Normal range < 4 mg/L. *N* = 132

### Biological measures

BDNF levels spanned from 13093 to 50434 pg/mL with a mean of 27184 pg/mL. Hs-CRP levels spanned from 0.0 to 77.1 mg/L with a mean of 3.5 mg/L, and median of 1.6 mg/L. When two outliers of hs-CRP > 35 mg/L were removed the median was reduced to 1.5 and the mean to 2.7 mg/L, Table 1.

### Psychotropic medication

Fourteen out of the 102 patients using regular psychotropic medication used a combination of either antidepressive medication and benzodiazepines/ Z-hypnotics or antidepressive medication and antipsychotic medication. 8 patients used a combination of antidepressive medication and benzodiazepines/ Z-hypnotics, and 6 used the combination of antidepressive and antipsychotic medication.

Since this is an inpatient ward for patients with depression and anxiety disorders and not for patients with psychotic disorders, we presume that the use of antipsychotic medication is off- label use to improve sleep and reduce agitation. Characteristics of study participants in the two different groups are presented in Tables 2 and 3.

**Table 2:** Characteristics of study participant with psychotropic medication

	Min	Max	Mean	SD
BMI	16	44	28	6
Age (years) BDNF (pg/ml) hs-CRP	18 13093 0.00	64 50434 14	38 27343 2.8	13 6158 3

BDNF: Normal range: 6186-42580 pg/mL. Hs-CRP: Normal range < 4 mg/L. *N* = 102

**Table 3:** Characteristics of study participant without psychotropic medication

	Min	Max	Mean	SD
BMI	15	55	29	10
Age (years) BDNF (pg/ml) hs-CRP	20 14004 0.0	78 42740 15	34 26647 2	14 6643 3

BDNF: Normal range: 6186-42580 pg/mL. Hs-CRP: Normal range < 4 mg/L. *N* = 30

### Psychometrics

Our participants had highest scores on depression, obsessive- compulsive, somatization, and anxiety symptom clusters in SCL-90, Table 4.

**Table 4:** Range of symptom clusters, raw-scores from SCL-90-R

	N^a^	Mean	Minimum	Maximum
Depression	125	27	0	46
Somatization	125	19	0	46
Anxiety cluster	126	17	0	37
Phobic anxiety	120	10	0	25
Paranoid ideation	122	7	0	20
Anger hostility	128	4	0	21
Psychotisism	127	9	0	30
Obsessive compulsive	126	20	0	38
Interpersonal sensitivity	126	16	0	35
Global severity index	127	12	0	22

^a^ Valid N 95, cases excluded listwise. Cases included in the analysis only if they have full data on all of the variables listed

### Multiple linear regression analysis

BDNF as well as hs-CRP were set as dependent variables and symptom clusters and confounding factors as predictors. We used split file for psychotropic medication and analysed in two subgroups; with or without psychotropic medication. In the regression analysis no co-linearity problem was detected, also evaluated with residual analysis.

In the subgroup of patients not using psychotropic medication, significantly higher BDNF was seen for all SCL-90 symptom clusters apart from anger-hostility, obsessive-compulsive and paranoid ideation. The results remained significant when adjusting for age, gender, smoking and BMI, Table 5.

**Table 5:** Multiple linear regression analysis of BDNF and symptom clusters from group with no psychotropic medication

	Coefficient	*P* - value*	95% CI
Depression	0.4	**0.032**	(115.8- 490.3)
Somatization	0.4	**0.028**	(49.4- 504.1)
Anxiety	0.6	**0.004**	(157.7- 641.8)
Phobic anxiety	0.4	**0.035**	(151.7- 770.9)
Paranoid ideation	0.3	0.175	(82.8- 872.2)
Anger hostility	0.4	0.066	(110.8- 1410.8)
Psychotisism	0.5	**0.031**	(163.2- 764.7)
Obsessive compulsive	0.3	0.073	(126.9- 658.3)
Interpersonal sensitivity	0.4	**0.025**	(161.1- 710.8)
Global severity index	0.5	**0.008**	(298.9- 1123.9)

*Adjusted for age, gender, smoking and BMI. BDNF dependent variable, symptom clusters independent variables

In the subgroup of patients using psychotropic medication the paranoid ideation cluster was the only symptom cluster associated with BDNF. The association was negative, adjusted for age, gender, smoking and BMI, table 6.

**Table 6:** Multiple linear regression analysis of BDNF and symptom clusters from group with psychotropic medication

	Coefficient	*P*-value*	95% CI
Depression	-0.03	0.750	(-136.1- 100.4)
Somatization	-0.10	0.357	(-163.2- 63.6)
Anxiety	-0.17	0.091	(-298.8- -0.9)
Phobic anxiety	-0.15	0.161	(-320.2- 42,7)
Paranoid ideation	-0.25	**0.022**	(-579.7- -39.2)
Anger hostility	-0.15	0.109	(-683.3- 22.2)
Psychotisism	-0.17	0.088	(-418.9- 9.8)
Obsessive compulsive	-0.17	0.094	(-281- 16.8)
Interpersonal sensitivity	-0.16	0.124	(-298.9- 4.8)
Global severity index	-0.18	0.081	(-467.0- 77.6)

*Adjusted for age, gender, smoking and BMI. Hs-CRP dependent variable, symptom clusters independent variables

For hs-CRP no associations with symptom clusters were seen in either group, adjusted for age, gender, smoking and BMI, Tables 7 and 8.

**Table 7:** Multiple linear regression analysis of hs-CRP and symptom clusters from group with no psychotropic medication

	Coefficient	*P*-value*	95% CI
Depression	0.1	0.724	-0.05- 3.9
Somatization	0.1	0.726	-0.1- 0.1
Anxiety	-0.1	0.639	-0.1- 0.1
Phobic anxiety	0.4	0.058	-0.2- 0.2
Paranoid ideation	0.3	0.275	-0.1- 0.3
Anger hostility	0.2	0.314	-0.2- 0.4
Psychotisism	0.2	0.390	-0.1- 0.2
Obsessive compulsive	-0.2	0.920	-0.1- 0.2
Interpersonal sensitivity	0.3	0.192	-0.1- 0.2
Global severity index	0.2	0.272	-0.2- 0.2

*Adjusted for age, gender, smoking and BMI. Hs-CRP dependent variable, symptom clusters independent variables

**Table 8:** Multiple linear regression analysis of hs-CRP and symptom clusters from group with psychotropic medication

	Coefficient	*P*-value*	95% CI
Depression	-0.03	0.752	-0.5- 0.1
Somatization	0.02	0.863	-0.3- o.1
Anxiety	0.09	0.396	-0.0- 0.1
Phobic -anxiety	-0.12	0.288	-0.1- 0.1
Paranoid ideation	-0.18	0.101	-0.1- 0.1
Anger hostility	-0.06	0.595	-0.1- 0.2
Psychotisism	-0.08	0.445	0.1-0.2
Obsessive compulsive	-0.17	0.100	-0.1- 0.1
Interpersonal sensitivity	-0.13	0.211	-0.1-0.1
Global severity index	-0.04	0.720	-0.1-0.2

* Adjusted for age, gender, smoking and BMI. Hs-CRP dependent variable, symptom clusters independent variables

## Discussion

Among patients not using psychotropic medications, significant positive associations were seen between BDNF and most symptom clusters; depression, somatization, interpersonal sensitivity, anxiety, phobic anxiety, and psychoticism, as well as a global severity index were found. The finding was consistent after correcting for confounders. For the patient group taking psychotropic medication an association between BDNF and symptom clusters was seen only for the paranoid ideation cluster. We did not find any association between hs-CRP and the different symptom clusters.

BDNF is suggested as a biomarker in mental disorders [16]. For the unmedicated patient group we found a positive association between level of BDNF and intensity of symptoms in several SCL-90 clusters. In line with our findings a recent study reported higher levels of BDNF in bipolar disorder, where higher levels of BDNF were associated with longer illness duration [17]. Some studies found significant positive association of BDNF with negative symptoms in schizophrenia and it has been suggested that the increased level of BDNF represents a compensatory response to the underlying neuropathology in the brain regions implicated [18]*.* Our findings also are in keeping with the findings of Kheirouri et al. [19] in patients with major depressive disorder (MDD).

However, most previous studies report a negative association between mental suffering and level of BDNF. This negative association is reported to reverse with successful treatment [5]*.* Some studies did not find any effect of medication on level on BDNF for depression symptom scores [16, 20]. For example, in one study olanzapine showed no alteration of BDNF after 8 weeks of treatment [21]. In another study BDNF was associated with specific personality traits and psychological job stress in Japanese employees [22]. In conclusion, results concerning BDNF levels in mental suffering are so far inconsistent. We speculate that our findings of significant positive associations between BDNF and most symptom clusters in non-medicated patients including depression, somatization, anxiety and psychoticism may be explained through the involvement of BDNF in the hypothalamic-pituitary-adrenal (HPA) axis in depression: The psychological stress of depression, causes an upregulated level of BDNF that in turn causes an increase in corticotrophin-releasing hormone leading to glucocorticoid release [19]. The findings however need to be repeated.

We did not find any association between increased symptoms and decreased BDNF in the group not taking medication, and only for one symptom cluster, Paranoid ideation, in the medication group. Evaluating this it is important to remember that at the time of assessment patients are expected to be improving from their mental suffering. One explanation may be that the body and brain conduct self-repair and compensates by upregulating BDNF. The group of patients using psychotropic medication did not show this upregulation despite the same symptom burden as reported by SCL-90-R.

We did not find any significant association between symptom clusters and hs-CRP when adjusted for confounding factors. Other studies have found that hs-CRP is related to severity of depressive symptoms [23]. Increased hs-CRP has been reported in patients with fibromyalgia and chronic fatigue syndrome [13] as well as in depression and anxiety [24]. We do not have a group of healthy controls and thus cannot claim that our patients actually have increased hs-CRP, but a mean level 2.7 still represent moderately increased risk of cardiovascular disease (low risk cut off < 1 mg/L).

Overall, we could not reproduce the findings from other studies in hs-CRP, and the findings on BDNF may seem in conflict with previous reports. Factors contributing to inconsistency in this field may be variations in methodology, symptom severity, medication, measure used to diagnose and size and ethnicity of the sample [25]. Previous studies have demonstrated associations between serum BDNF and lifestyle factors as smoking, BMI and physical activity [26], as well use of psychotropic medication [5]. It is also known that age and gender can affect the BDNF level. A higher BDNF serum level is associated with increasing age, residence in high degree of urbancy and smoking [25].

There are several limitations to the study. Information on symptoms, smoking, weight and height in our study were obtained from the self-report questionnaire with a risk of biased reporting. The sample size is small, especially regarding the subgroup with no use of psychotropic medication. However, other similar studies published in this field report smaller numbers (*n* < 100) [16, 19, 25]. Our aim was to search for association between BDNF and mental suffering in the two groups; with or without psychotropic medication. We did not study BDNF in relation to diagnoses or symptoms compared to a healthy control group, and this is a main limitation. However, we are still able to compare general mental suffering with BDNF and hs-CRP adjusting for the known confounding factors. We do not use psychiatric diagnostic groups in this study, thus we cannot relate the findings directly to other studies based on diagnostic groups. Also, this is a cross sectional natural study. Patients were not randomised to either medication or non-medication. Furthermore, as mentioned they were not acutely ill. Another limitation might be that presence of other physical illnesses were not considered.

One main originality of our study is the focus on symptoms and symptom clusters rather than diagnostic groups. Another original factor in our study is that the data were sampled roughly 7 days after admittance to reduce potential stress achieved by the admittance itself for the patients and thereby to reduce any possible stress-induced alterations in the biological markers. During these 7 days patients will also have been exposed to care, safety, professional milieu therapy, psychotherapy, often improved sleep and nutrition. All of which could contribute to reduce the possible stress of admittance to the inpatient setting. Also, the focus on a general open ward inpatient group may add information to the field regarding effect of different degrees of illness compared to patient groups in other studies.

There also are several general strengths. We adjusted for smoking, weight and height as well as use of psychotropic medication. Regarding biological measures, serum was sampled after rest and fasting in the morning to avoid effects of diurnal variation. As serum BDNF levels have been shown to be significantly lower if blood is drawn in the afternoon and diurnal variations of BDNF plasma levels are known [26] we sampled sera fasting in the morning, and without previous exercise. Furthermore, the laboratory used a standardized protocol for analysis and with an experienced technician performing the analysis.

## Conclusion

Our findings revealed that contrary to most previous reports, serum BDNF levels were positively associated with several psychiatric symptoms in the group of patients not using psychotropic medication. This was not seen in the group on psychotropic medication. We speculate that the group of patients not using psychotropic medication depict more biological alteration than those on medication, and that upregulation of BDNF has occurred in the medication free group. Patients using psychotropic medication are probably not in need of the same upregulation because the medication are modulating the underlying biological pathology, but this needs further investigation.

Hs-CRP had a mean level of 2.7 mg/L which may represent a moderately increased risk of cardiovascular disease [27], but no associations were found to the symptom clusters. This is in line with the findings in other studies that increased hs-CRP is not specific for a certain symptom cluster, but rather related to mental suffering and cardiovascular risk in general.

The major contribution of the present study is the use of symptoms rather than diagnosis, the integration of confounding factors, and standardized analysis of serum BDNF. To our knowledge this is the only report on the relationship between psychiatric symptoms, BDNF and hs-CRP in two groups; with and without psychotropic medication use in a sample with inpatients with general mental disorders from Northern Norway.

Our results provide further evidence for a role for BDNF in the pathophysiology of mental disorders, especially revealed in the group of patients not using psychotropic medication. Furthermore, it also suggests that BDNF alteration in general mental disorder reflect response to general stress and mental illness, independent of presented symptoms.

## Data Availability

Our dataset contains individual personalized information such as date of birth and zip codes, and thus cannot be shared but it can be available from the corresponding author on reasonable request.

## Abbreviations

BDNF: Brain Derived neurotropic Factor
BMI: Body Mass Index
CI: Confidence interval
hs-CRP: High sensitive C-Reactive protein
SCL-90-R: Symptom Check List Revised
SPSS: Statistical Package for the Social Sciences

## References

[1] Toll A, Mane A (2015). Brain-derived neurotrophic factor levels in first episode of psychosis: a systematic review. World J Psychiatry.

[2] Pan W, Banks WA, Fasold MB, Bluth J, Kastin AJ (1998). Transport of brain-derived neurotrophic factor across the blood-brain barrier. Neuropharmacology.

[3] Karege F, Schwald M, Cisse M (2002). Postnatal developmental profile of brain-derived neurotrophic factor in rat brain and platelets. Neurosci Lett.

[4] Pillai A, Kale A, Joshi S, Naphade N, Raju MS, Nasrallah H, Mahadik SP (2010). Decreased BDNF levels in CSF of drug-naive first-episode psychotic subjects: correlation with plasma BDNF and psychopathology. Int J Neuropsychopharmacol.

[5] Nomoto H, Baba H, Satomura E, Maeshima H, Takebayashi N, Namekawa Y, Suzuki T, Arai H (2015). Serum brain-derived neurotrophic factor levels and personality traits in patients with major depression. BMC Psychiatry.

[6] Fernandes BS, Berk M, Turck CW, Steiner J, Goncalves CA (2014). Decreased peripheral brain-derived neurotrophic factor levels are a biomarker of disease activity in major psychiatric disorders: a comparative meta-analysis. Mol Psychiatry.

[7] Kim JR, Kim HN, Song SW (2018). Associations among inflammation, mental health, and quality of life in adults with metabolic syndrome. Diabetol Metab Syndr.

[8] Sproston NR, Ashworth JJ (2018). Role of C-reactive protein at sites of inflammation and infection. Front Immunol.

[9] Bebbington P (2015). Categories, continua and the growth of psychiatric knowledge. Soc Psychiatry Psychiatr Epidemiol.

[10] Hyman SE (2011). Grouping diagnoses of mental disorders by their common risk factors. Am J Psychiatry.

[11] Lakhan SE, Vieira K, Hamlat E (2010). Biomarkers in psychiatry: drawbacks and potential for misuse. Int Arch Med.

[12] Oiesvold T, Nivison M, Hansen V, Skre I, Ostensen L, Sorgaard KW (2013). Diagnosing comorbidity in psychiatric hospital: challenging the validity of administrative registers. BMC Psychiatry.

[13] Groven N, Fors EA, Reitan SK (2019). Patients with fibromyalgia and chronic fatigue syndrome show increased hsCRP compared to healthy controls. Brain Behav Immun.

[14] Larsen JB, Stunes AK, Vaaler A, Reitan SK (2019). Cytokines in agitated and non-agitated patients admitted to an acute psychiatric department: a cross-sectional study. PLoS One.

[15] Schmitz N, Hartkamp N, Kiuse J, Franke GH, Reister G, Tress W (2000). The symptom check-list-90-R (SCL-90-R): a German validation study. Qual Life Res.

[16] Skibinska M, Kapelski P, Rajewska-Rager A, Pawlak J, Szczepankiewicz A, Narozna B, Twarowska-Hauser J, Dmitrzak-Weglarz M (2018). Brain-derived neurotrophic factor (BDNF) serum level in women with first-episode depression, correlation with clinical and metabolic parameters. Nord J Psychiatry.

[17] Munkholm K, Vinberg M, Kessing LV (2016). Peripheral blood brain-derived neurotrophic factor in bipolar disorder: a comprehensive systematic review and meta-analysis. Mol Psychiatry.

[18] Binford SS, Hubbard EM, Flowers E, Miller BL, Leutwyler H (2018). Serum BDNF Is positively associated with negative symptoms in older adults with schizophrenia. Biol Res Nurs.

[19] Kheirouri S, Noorazar SG, Alizadeh M, Dana-Alamdari L (2016). Elevated brain-derived neurotrophic factor correlates negatively with severity and duration of major depressive episodes. Cogn Behav Neurol.

[20] Atake K, Yoshimura R, Hori H, Katsuki A, Ikenouchi-Sugita A, Umene-Nakano W, Nakamura J (2014). Duloxetine, a selective noradrenaline reuptake inhibitor, increased plasma levels of 3-methoxy-4-hydroxyphenylglycol but not homovanillic acid in patients with major depressive disorder. Clin Psychopharmacol Neurosci.

[21] Hori H, Yoshimura R, Yamada Y, Ikenouchi A, Mitoma M, Ida Y, Nakamura J (2007). Effects of olanzapine on plasma levels of catecholamine metabolites, cytokines, and brain-derived neurotrophic factor in schizophrenic patients. Int Clin Psychopharmacol.

[22] Okuno K, Yoshimura R, Ueda N, Ikenouchi-Sugita A, Umene-Nakano W, Hori H, Hayashi K, Katsuki A, Chen HI, Nakamura J (2011). Relationships between stress, social adaptation, personality traits, brain-derived neurotrophic factor and 3-methoxy-4-hydroxyphenylglycol plasma concentrations in employees at a publishing company in Japan. Psychiatry Res.

[23] Kohler-Forsberg O, Buttenschon HN, Tansey KE, Maier W, Hauser J, Dernovsek MZ, Henigsberg N, Souery D, Farmer A, Rietschel M (2017). Association between C-reactive protein (CRP) with depression symptom severity and specific depressive symptoms in major depression. Brain Behav Immun.

[24] Tayefi M, Shafiee M, Kazemi-Bajestani SMR, Esmaeili H, Darroudi S, Khakpouri S, Mohammadi M, Ghaneifar Z, Azarpajouh MR, Moohebati M (2017). Depression and anxiety both associate with serum level of hs-CRP: a gender-stratified analysis in a population-based study. Psychoneuroendocrinology.

[25] Elfving B, Buttenschon HN, Foldager L, Poulsen PH, Andersen JH, Grynderup MB, Hansen AM, Kolstad HA, Kaerlev L, Mikkelsen S (2012). Depression, the Val66Met polymorphism, age, and gender influence the serum BDNF level. J Psychiatr Res.

[26] Bus BA, Molendijk ML, Penninx BJ, Buitelaar JK, Kenis G, Prickaerts J, Elzinga BM, Voshaar RC (2011). Determinants of serum brain-derived neurotrophic factor. Psychoneuroendocrinology.

[27] Ridker PM, Rifai N, Rose L, Buring JE, Cook NR (2002). Comparison of C-reactive protein and low-density lipoprotein cholesterol levels in the prediction of first cardiovascular events. N Engl J Med.

